# Sensory Stimulation-Based Complete Protection from Ischemic Stroke Remains Stable at 4 Months Post-Occlusion of MCA

**DOI:** 10.4172/2329-6895.1000135

**Published:** 2013-10-05

**Authors:** Aneeka M Hancock, Christopher C Lay, Melissa F Davis, Ron D Frostig

**Affiliations:** 1 Department of Neurobiology and Behavior, University of California, Irvine, California, USA; 2 Department of Biomedical Engineering, University of California, Irvine, California, USA; 3 The Center for the Neurobiology of Learning and Memory, University of California, Irvine, California, USA; 4 The Center for Hearing Research, University of California, Irvine, California, USA

**Keywords:** Ischemia, Imaging, Chronic, Stroke, Animal models, Brain recovery

## Abstract

Previous research from our lab has shown that when using a rodent model of ischemic stroke (permanent middle cerebral artery occlusion), mild sensory stimulation, when delivered within two hours of ischemic onset, completely protects the cortex from impending ischemic stroke damage when assessed 24 hours post-occlusion. However, the long-term stability of this protection remains unclear. Using intrinsic signal optical imaging for assessment of cortical function, laser speckle imaging for assessment of blood flow, a battery of behavioral tests and cresyl violet for histological assessment, the present study examined whether this protection was long-lasting. When assessed 4 months post-occlusion (this length of time being equivalent to 10–15 years in humans), rats receiving sensory stimulation treatment immediately after ischemic onset exhibit normal neuronal and vascular function, and they are behaviorally and histologically equivalent to healthy controls (surgical shams). Thus, the complete neuroprotection due to cortical activation via sensory stimulation remains stable with time. These findings add support to the translational potential of this sensory stimulation-based treatment.

## Introduction

Stroke is the fourth leading cause of death in the United States, and is also a leading cause of long-term disability, with annual direct and indirect costs nearing 40 billion dollars [1]. The aftermath of a stroke can include hemiparesis, cognitive deficits, depression, dependency on others for daily living, aphasia, and even institutionalization [1,2]. Despite the fact that numerous neuroprotective therapies have been tested in rodents over the past 20 years, none have resulted in improved outcome in phase III clinical trials [3]. Currently, the only FDA approved drug for ischemic stroke is recombinant tissue plasminogen activator (rt-PA), which can only be given to certain subgroups of patients [4], if the patient quickly arrives at the hospital after the incident and suffers from an ischemic event [5,6], which comprises 87% of all strokes [1]. Even then, this drug can have harmful side effects [6]. Clearly, there is a need for a rapid and long-lasting treatment to protect from stroke damage.

We have previously demonstrated that a form of mild sensory stimulation, intermittent mechanical single-whisker stimulation, when delivered immediately (within 1 hour, and in most cases within 2 hours) after permanent middle cerebral artery occlusion (pMCAO), completely protects rodent cortex from impending functional and structural ischemic stroke damage [7] (reviewed in [8]). Treatment consisted of 4.27 minutes of 1-second, 5-Hz, 9° deflections of a single whisker intermittently during a 120-minute treatment period. Utilizing multiple techniques, such as functional imaging, blood flow imaging, electrophysiological recording, behavioral assessment, and histology, we have confirmed that this mild stimulation results in the gradual recovery of cortical function and reperfusion of the MCA via collateral vessels during the treatment period itself [7,9]. Functional imaging, blood flow imaging, and neuronal recordings showed that cortical function was at or above baseline levels at 24 hours post-pMCAO, while behavioral assessment at 7 days post-pMCAO revealed that rats had no sensorimotor deficits, and histological analysis at 24 hours and 7 days post-pMCAO showed no infarct [7]. This protection has been observed in young adult rats (3–4 months of age), as well as in aged rats (21–24 months of age) [7,9,10]. Non-stimulated control subjects, those that received the pMCAO but no whisker stimulation, showed reduced whisker representations with functional imaging (ISOI), and sustained infarcts according to TTC staining, when assessed 24 hours post-occlusion.

A pivotal question related to the translational potential of these findings is whether this complete protection from impending stroke damage is present for only a short duration, or whether it is truly long-lasting, especially given the major neurovascular plasticity that occurred in these animals enabling reperfusion of the ischemic area. Namely, following whisker stimulation, blood flows in reverse of its normal direction within the permanently occluded MCA, a flow originating from collateral vessels [7]. To address this question, we assessed rats 4 months post-pMCAO to determine whether cortical function remained intact in rats that received immediate post-occlusion whisker stimulation. Accordingly, we focused solely on protection conferred by whisker stimulation, rather than recovery from ischemic damage in non-stimulated controls, which is qualitatively a different study. Employing functional imaging, blood flow imaging, behavioral assessment and histology, we demonstrate that in rats receiving a middle cerebral artery occlusion followed immediately by whisker stimulation (+0h subjects, meaning zero hours between time of occlusion and onset of stimulation), cortical function remains intact, blood flow stable, structure undamaged, and behavioral measures are normal compared to a sham-surgery control group, when assessed at 4 months post-pMCAO. Given that 4 months in rats is a significant portion of their lives, equivalent to 10–15 years in humans [11], the presence of intact cortical function and structure in the +0h subjects at 4 months post-occlusion suggests that this treatment results in a quick and stable protection from ischemic damage following pMCAO.

## Methods

All procedures were in compliance with NIH guidelines and approved by UC Irvine Animal Care and Use Committee (protocol #: 1997-1608, assurance ID#: A3416.01).

### Subjects and surgical preparation

Twenty-four experimental subjects, 295–400 g (3–4 months of age) male Sprague Dawley rats (Charles River Laboratories, Wilmington, MA, USA), were individually housed in standard cages. At the beginning of each experiment, subjects were injected intraperitoneally with a Nembutal bolus (55 mg/kg b.w.). Supplemental injections of Nembutal (27.5 mg/kg b.w.) were given as necessary. After resection of soft tissue, a ~6.5×8 mm ‘imaging’ area of the skull over the left primary somatosensory cortex (rostromedial corner positioned approximately 1 mm caudal and 2 mm lateral from bregma) was thinned to ~150 μm using a dental drill. Five percent dextrose (3 mL) and atropine (0.05 mg/kg, b.w.) were administered at the beginning of the experiment and every six hours after until the animal was returned to its home cage (the first day of each experiment typically lasted 8 to 10 hours and the second day, at 4 months post-occlusion, typically lasted 6 to 8 hours). Body temperature was measured via a rectal probe, and maintained at 37° Celsius by a self-regulating thermal blanket. After the completion of the experiment, all animals were returned to their home cage and allowed to recover. All subjects received flumeglumine (2.5 mg/kg b.w.) at the end of surgery, and the health of the animals were monitored daily until their 4 month assessment. Animals remained housed in their home cage throughout the 4-month period.

### Overview

**Figure 1:**
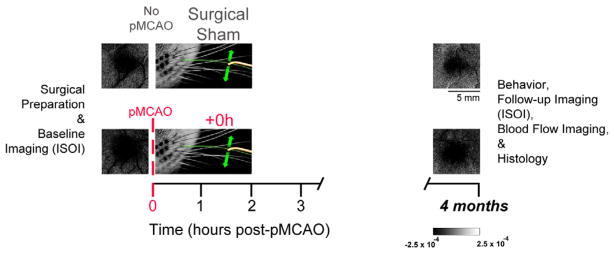
Experimental timeline and representative examples of functional imaging findings. Rats underwent baseline ISOI, followed by either a sham occlusion (surgical sham subjects), or a pMCAO (+0h subjects). All rats then received 2 hours of intermittent C2 whisker stimulation treatment. Four months later, all subjects underwent follow-up ISOI, as well as behavioral assessment, bloodflow imaging and histology. Images on far left and right are of the ipsi-ischemic C2 whisker functional representation collected before and 4 months after pMCAO. Linear grayscale bar indicates intrinsic signal strength ×10^−4^. Black and white streaks correspond to large surface blood vessels.

Functional imaging, blood flow imaging, and behavior timelines are summarized in Figure 1. Using a within subject design that is identical to our previous studies, 24 subjects were randomly assigned to a +0h group or a sham surgical control group. Baseline functional imaging was collected for all subjects at the beginning of surgery. All +0h subjects (n=12) then received a pMCAO, and immediate post-occlusion whisker stimulation. Post-occlusion whisker stimulation consisted of 1 s of 5 Hz deflections of a single whisker (whisker C2). This stimulation was intermittently (with random intervals averaging 21 seconds) delivered 256 times, totaling 4.27 minutes of stimulation, over the course of 2 hours [7]. Surgical shams (n=12) underwent identical surgery to that of +0h subjects, with the suture needle and thread passing under the MCA, but sutures were not tied around the MCA, leaving the blood vessel intact. Sham surgery was immediately followed by whisker stimulation. After whisker stimulation, all rats were placed back in their home cage for recovery, until 1 to 3 days before their assessment 4 months later, at which point behavioral health was evaluated. For the 4 month assessment, functional imaging, followed by blood flow imaging, was conducted. Rats were then transcardially perfused and brains were sectioned for cresyl violet staining (See below for detailed methodology and experimental design).

### Permanent middle cerebral artery occlusion (pMCAO)

Ischemic conditions were achieved via surgical occlusion of the stem of the left proximal middle cerebral artery [12–14]. The skull and dura were carefully removed from a 2×2 mm ‘surgical window’ just anterior and lateral to the imaging window (over MCA’s stem, also known as the M1 segment just distal to MCA’s lenticulostriate branch) and a half-curve reverse cutting suture needle and thread (4-0 silk) was passed through the pial layer of the meninges, below MCA and above the cortical surface. To ensure that our pMCAO had completely and permanently obstructed blood flow, we performed a double ligature technique and transection of the MCA, the details of which are outlined by Davis et al. [15]. This preparation did not change for the entire 4 months of the experiment.

### Histology

Concluding the 4 month assessment, rats were perfused transcardially with PBS, 1% gelatin, and 4% paraformaldehyde [12]. Their brains were carefully removed, then post-fixed overnight, and placed in 30% sucrose until ready for sectioning. Brains were embedded in tissue-freezing medium for cryostat sectioning, and were coronally sectioned at 40 μm. Every 5^th^ section was mounted on a slide, stained with cresyl violet, and coverslipped with mounting medium. Images of each section were captured with a 1.5× objective. The Paxinos and Watson rat brain atlas was used to identify anatomical structures [16]. A small surgical lesion (~1 mm in diameter) was occasionally apparent at the immediate site of MCA occlusion. This occurred infrequently and equivalently in both +0h and surgical sham groups.

### Intrinsic signal optical imaging (ISOI) and analysis

A detailed description of ISOI [17–20] data acquisition and analysis can be found elsewhere [21,22]. Briefly, a charge-coupled device (CCD) camera (a 12-bit Quantix 0206) equipped with an inverted 50 mm AF Nikon lens (1:1:8) combined with an extender (model PK-13) was used for imaging and controlled by V++ Precision Digital Imaging System software (Digital Optics). The cortex was illuminated with a red light-emitting diode (635 nm maximum wavelength with full width at half height of 15 nm). During each 15-second trial, 1.5 seconds of prestimulus data followed by 13.5 seconds of poststimulus onset data were collected, with a 6 ± 5-second random intertrial interval. Stimulus consisted of a single whisker (whisker C2) being deflected by ~9° in the rostral–caudal direction at a rate of 5 Hz for a total stimulus duration of 1 second. Data was collected in blocks of 64 stimulation trials over periods of about 30 minutes each. Ratio images were created from calculating fractional change values for each of the four 64 trial blocks by dividing each 500 ms frame of poststimulus signal activity by the 500 ms frame of prestimulus intrinsic signal activity collected immediately before stimulus onset [23]. The first phase of the evoked functional representation, the initial dip, was analyzed. This phase is generally associated with the evoked neural activity due to stimulation of a single whisker. The ratio image containing the maximal areal extent for this phase was Gaussian filtered, and the areal extent was quantified at a threshold level of 2.5×10^−4^ fractional change away from zero. Peak amplitude was quantified in fractional change units of the peak activity pixel for this intrinsic signal phase.

### LSI of blood flow and analysis

A detailed description of LSI [24,25] data acquisition and analysis can be found elsewhere [7]. Briefly, a 632.8 nm 15 mW HeNe laser was used as the illumination source. The speckle pattern from the 5.12×5.12 mm imaged region was captured as 512×512 pixel images by a 16-bit CCD camera (Cascade 512F) equipped with a Navitar zoom lens plus extenders such that speckle size matched camera pixel size. Collected images were processed as previously described [7]. Speckle contrast images were converted to speckle index images by calculating their inverse squares multiplied by the exposure time in seconds, so that larger index values corresponded to faster blood flow. Speckle index images were then averaged to improve signal-to-noise ratio. To quantify blood flow within the MCA, we calculated the mean value within a region of interest (ROI) in MCA cortical branches as defined according to several criteria described previously [7]. All flow index values were scaled over a range where 0 flow was set at noise values. Dead animal (noise) values were subtracted from all values.

### Behavioral tests

Sensorimotor behavior was assessed at 4 months post-MCAO to evaluate neurological health and determine if any ischemic damage had rendered the rats impaired. All behavioral testing occurred one to three days prior to all 4 month imaging and perfusion. Bederson neurological scores [26] were assigned to each rat to assess the general mobility of subjects, and whisker- and forepaw-guided behavior was assessed as previously performed [7]. Briefly, forepaw-guided exploration was assessed by placing subjects in the center of a testing cylinder (20 cm in diameter and 45 cm in height) for five minutes, during which initiation of a wall touch was scored, following rearing using the left forepaw, right forepaw, or both paws together. Wall touches were calculated, and forepaw use was expressed as a forepaw asymmetry score (right paw touches minus left paw touches), with a negative score signifying a subject’s preference to explore with the left forepaw. In normal subjects, there is a roughly even distribution of usage between left and right paws, while unilateral damage to the somatosensory cortex will result in a greater dependence upon the unaffected limb [27].

Whisker-guided exploration was assessed by placing each subject in a 25-cm-wide rectangular track (120×80 cm, outer diameter) and was allowed 10 s to acclimate before the start of the 5 minute testing session. Whisker scanning was defined as the time spent by the subject touching the walls of the rectangular track with one set of whiskers while locomoting [23]. Care was given to exclude incidents such as rearing and grooming as well as exploration which involved scanning with both sets of whiskers simultaneously, as in the case when a rat is facing perpendicular to a wall surface. Scanning was measured in seconds spent using either the left or right whisker pad by a timer watching the recorded testing session. Each subject was then assigned a thigmotactic scanning score (right score minus left score), with a negative score signifying a subject’s preference to scan with the left set of whiskers. While healthy animals occasionally exhibit a whisker set preference, averages across groups of animals do not suggest a disproportionate preference for one whisker set over the other. Animals with unilateral damage to the somatosensory cortex, however, show a preference only for the unaffected whisker set [28]. Observers blind to the rats’ experimental conditions performed all behavioral data analysis.

### Statistical analysis

Inferential statistics were performed on the raw values of ISOI data, laser speckle velocity and all behavioral data. For ISOI analysis, a repeated measures ANOVA with one between subjects variable (experimental group, +0h vs. sham) and one within subjects variable (time, baseline vs. 4 months) was performed, followed by *post hoc* contrasts to identify which post-occlusion values were significantly different from baseline. Alpha level was set to 0.05 and Bonferroni adjustments were applied to account for multiple contrasts (2 contrasts for an adjusted alpha value of 0.025). For LSI, and forepaw- and whisker-guided behavior, two-sample unpaired t-tests were performed with an α-level of significance set at 0.05. Fisher’s exact test was performed for Bederson scores. All plotting and statistics were performed using SYSTAT 11 (SYSTAT Software Inc., Chicago, IL, USA).

## Results

### Cortical function remains stable 4 months post-pMCAO when followed by immediate stimulation treatment

**Figure 2:**
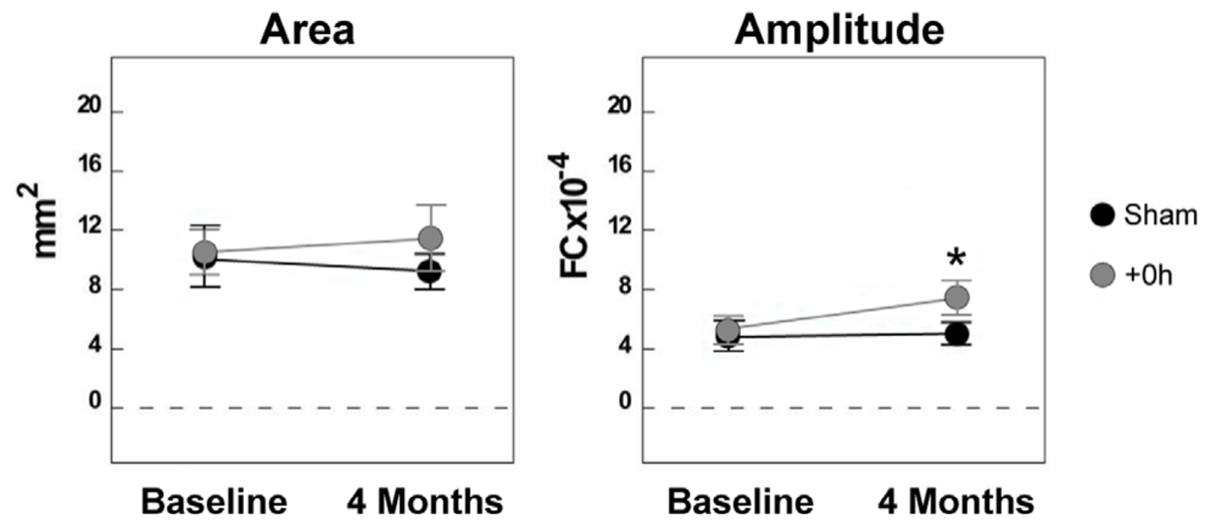
Cortical function remains stable at 4 months post-pMCAO. In each graph, group baseline is paired with 4 month data. Means and standard errors are provided for the area and amplitude of the evoked functional representation from the stimulation of the contra-ischemic C2 whisker before and 4 months after pMCAO. A value of zero indicates no response to whisker stimulation. Asterisk indicates significant difference between +0h and sham subjects at 4 months (p=0.006).

To determine whether this sensory stimulation-based treatment conferred complete, long-lasting protection from ischemic stroke, we first wanted to know if cortical function remained intact at 4 months post-occlusion of MCA. To assess cortical function, we collected baseline ISOI data. Rats were randomly assigned to one of two experimental groups after baseline imaging. Subjects in the +0h group (n=12) received a MCA occlusion followed immediately by whisker stimulation, while subjects in the surgical sham group (n=12) underwent surgery (leaving the MCA intact), then immediately received whisker stimulation. Four months post-occlusion, the rats underwent imaging again. ISOI revealed no change in the area or amplitude after 4 months in surgical shams. Normal cortical activity was observed in +0h subjects compared to surgical shams that never received the occlusion (Figure 2), evidenced by the fact that the area and amplitude of the functional representation did not decrease below sham values. In fact, the area and the amplitude of the initial dip were increased in +0h subjects at 4 months compared to their baseline, with the amplitude nearly having a significant increase at 4 months compared to baseline (p=0.03, not significant with Bonferroni adjustment), but a significant increase in +0h subjects at 4 months compared to shams (p=0.006). Interestingly, these trends for the increase in area and amplitude in +0h subjects at 4 months compared to baseline, and the significant increase in amplitude at 4 months in +0h subjects compared to shams, are similar to our previous findings of the same parameters for the initial dip when assessed 24 hours post-occlusion [7].

**Figure 3:**
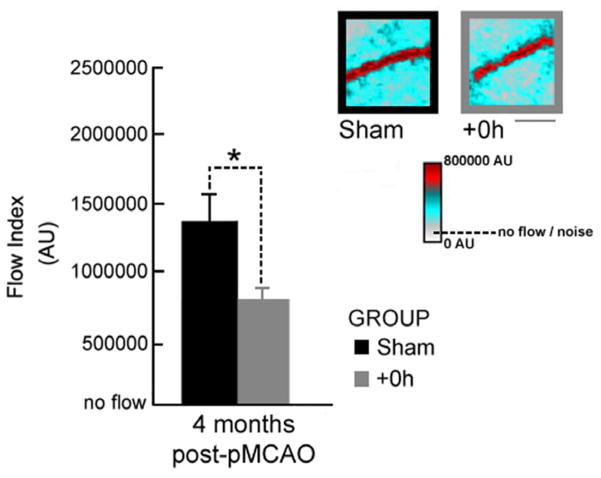
LSI demonstrates that at 4 months post-pMCAO, blood flow is maintained in the occluded MCA. Insets, representative linearly color scaled LSI images of the MCA taken at 4 months post-pMCAO for a surgical sham and +0h subject. Blood flow is apparent in both surgical sham and +0h subjects. Flow is expected in shams since the MCA remained intact, while reperfusion of the MCA is maintained in +0h subjects. Scale bar indicates 0.25 mm. Graph, the x-axis crosses the y at the mean noise level, or, ‘no flow’ level. Means and standard errors for MCA flow at 4 months post-pMCAO. Asterisk indicates a significant difference between flow in surgical shams and +0h subjects at 4 months (p=0.0004).

Blood flow was assessed with laser speckle imaging (LSI). Given that the MCA was completely and permanently occluded in the +0h subjects, we wanted to confirm that the reversal of blood flow through the occluded MCA that we observe at 24 hours [7] was still present at 4 months post-occlusion. Our analysis showed that this reperfusion of the MCA still exists at 4 months post-occlusion and, surprisingly, also revealed a significant difference between the surgical shams and the +0h subjects (p = 0.0004) at this time point (Figure 3).

### Stimulation treatment results in normal sensorimotor-related behaviors at 4 months

**Figure 4:**
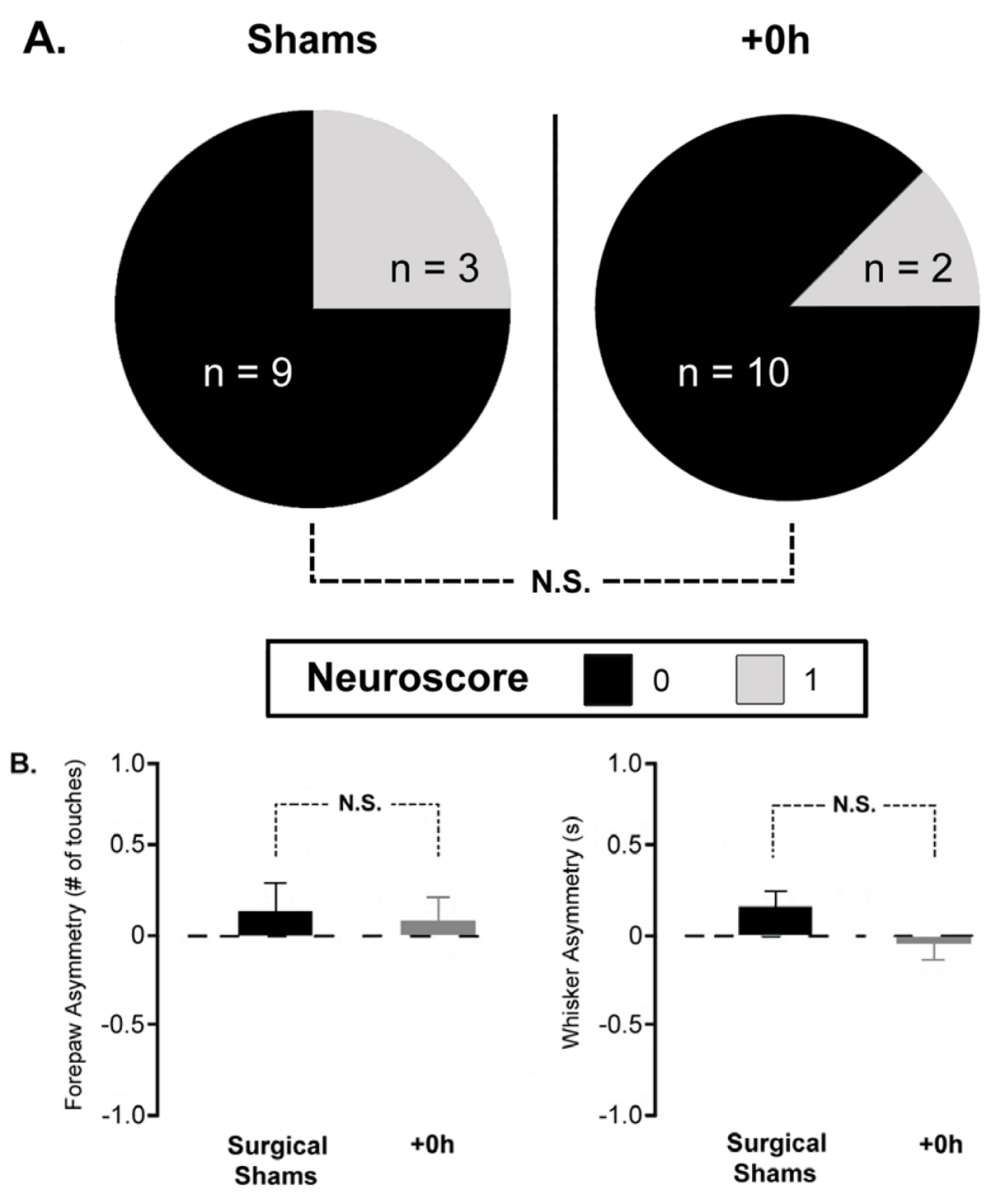
Whisker stimulation treatment results in normal sensorimotor behavior at 4 months. A, Neuroscores according to the Bederson scale for sham and +0h subjects. Pie charts represent the number of rats with the corresponding neurological score. All subjects had a score of 0 or 1, with no significant difference between sham and +0h groups. B, Forepaw-guided (left) and whisker-guided (right) asymmetry scores 4 months after pMCAO. Horizontal line indicates “0,” or no asymmetry. No significant difference exists between shams and +0h subjects for either type of exploration. All analysis was conducted by blind observers.

Previously, behavioral assessments at 7 days post-pMCAO reveal no sensorimotor impairments [7]. In order to determine whether the rats had any sensorimotor-related abnormalities at 4 months post-occlusion, each animal underwent the same behavioral tests as in our previous study: assessment according to the Bederson neurological scale, as well as with forepaw- and whisker-guided exploration. For the Bederson score, rats were assessed for the presence of limb flexion during suspension, a recognized sign of ischemic injury, as well as spontaneous circling behavior, difficulty with gait, and difficulty remaining upright while placed in a large cylindrical chamber and allowed to roam freely for five minutes. Results were then scored on a 0–4 scale, with 0 representing normal movement, and 4 representing a complete lack of spontaneous movement or stupor [12]. +0h subjects demonstrated unimpaired behavior on all tasks (Figure 4). No significant difference from surgical shams was observed according to the Bederson scores (p=0.25), where all subjects in both groups had a score of either 0, or a 1 (indicating the presence of limb flexion or circling behavior, but not both). Additionally, asymmetry scores for whisker- (p=0.14) and forepaw-guided exploration (p=0.71) showed equivalent performance for sham and +0h subjects.

### +0h subjects remain anatomically intact and histologically equivalent to shams at 4 months post-occlusion

**Figure 5:**
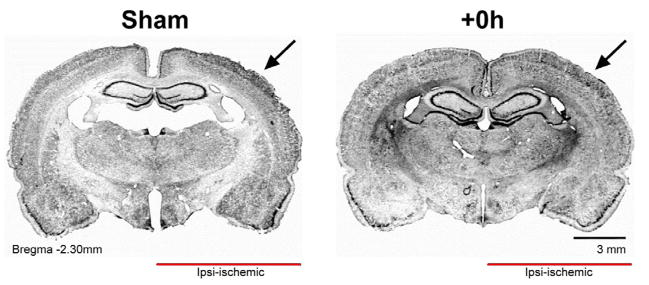
Cortical structure in +0h subjects remains equivalent to surgical shams at 4 months post-pMCAO. Representative coronal sections showing primary somatosensory cortex in sham and +0h subjects. Arrows point toward MCA blood supply territory for this cortical region. Cresyl violet staining shows no glial scarring in +0h subjects.

Given that there is a large influx of astrocytes and microglia into the region of infarct as the glial scar forms after an ischemic insult [29–31], the routinely used method of determining stroke-related lesions with 2% 2,3,5-triphenyltetrazolium chloride [32,33] could not be utilized here, as this method relies on functioning mitochondria to stain healthy tissue red, and glia could therefore show a false positive for healthy cortical neurons. Thus, we employed another widely utilized stain, cresyl violet, to resolve healthy tissue from any glial scar that could be present at 4 months post-occlusion [32] (Figure 5). Subjects served as internal controls. Histological analysis revealed no glial scar or other ischemic damage, such as abnormal cortical anatomy, in the ipsi-ischemic hemisphere of +0h subjects at 4 months, as compared to the contra-ischemic hemisphere. Additionally, +0h subjects were identical to surgical shams, with no evidence of ischemic damage present.

## Discussion

Utilizing a rat model of ischemic stroke and a battery of techniques, such as functional imaging, blood flow imaging, behavioral tests and histology, this study demonstrates that the complete protection of cortical function and structure observed 24 hours after pMCAO remains stable even 4 months after a pMCAO. To our knowledge, this is the first time neuroprotection from ischemic damage in rodents has been observed over such a long period of their lives, and has been assessed with multiple measures of cortical function, structure, and health.

One interesting finding arose from the ISOI analysis: the initial dip data for area and amplitude from +0h subjects and shams is similar at both 24 hours [7] and 4 months. This alone indicates that the protection of cortical function that exists early on in these +0h subjects remains out to 4 months. At 24 hours post-occlusion, we observe a significant increase in the amplitude of the initial dip compared to baseline [7]. Although that significance is not present at 4 months, there still exists an increase in +0h subjects at 4 months when compared to baseline, and a significant increase in amplitude when compared to shams at 4 months. This amplitude increase at 24 hours and 4 months compared to baseline possibly represents underlying neuroprotective plastic changes that seem to be maintained, at least when compared to sham controls.

We have demonstrated in our previous studies that the protection observed at 24 hours is due to a massive reorganization of blood flow in the MCA, with blood flowing backwards into the occluded MCA in order to continue reperfusing the ischemic cortical tissue, and that this reversal of flow occurs during the 2 hour treatment period [7,9]. It is possible that this could have been an acute response to the ischemic insult, with more metabolically demanding processes, such as angiogenesis, compensating for reduced blood flow in the long run [34,35]. However, we did not detect any overt angiogenesis in our +0h subjects at 4 months, but did observe a maintenance of blood flow in the occluded MCA. Not only did we see normal blood flow in +0h subjects, but surgical shams exhibited increased blood flow compared to +0h subjects. Given that this reperfusion of the MCA is constrained by the size of collateral vessels [7], it’s possible that this collateral reverse flow is slower in +0h subjects when compared to flow in the intact MCA of surgical shams where blood flow was never impeded.

In addition to cortical function remaining fully protected, and reversed blood flow being maintained in the MCA at 4 months, the +0h subjects were behaviorally and histologically equivalent to surgical shams. Many stroke patients suffer from debilitating, and long-lasting effects of ischemia, and despite some patients’ recovery over time with rehabilitation, many still do not make a full recovery and can have lifelong damage to their brain. Glial scarring and loss of brain tissue are common after an ischemic event, and can be detected histologically years after the occlusion [36,37]. In our +0h subjects, no evidence of ischemic damage or glial scarring was found, which would appear as a more densely-stained region due to the high density of glia in the infarct region [38]. Behaviorally, +0h subjects displayed no preference for an unaffected whisker pad or limbs, and no other sensorimotor deficits were detected, complementing the imaging, blood flow, and histological findings.

In conclusion, the ideal stroke treatment would not only be rapid, but also long-lasting. The sensory stimulation-based treatment herein fits this description. If initiated immediately after ischemic onset, mild sensory stimulation, a no side-effects treatment, confers complete protection from ischemic stroke in rats, and remains stable over a significant portion of the rats’ lifetime. As with the many neuroprotective treatments that have shown promise over the years, it is possible that the phenomenon observed here might be due to unique characteristics of the rodent brain and physiology. Thus, caution should be taken with any new potential treatment for stroke. Nevertheless, this study, involving a new type of neuroprotective treatment for stroke, serves to further highlight the translational potential of this sensory stimulation as a means of neuroprotection from ischemic stroke in humans.

## Abbreviations

rt-PA: Recombinant Tissue Plasminogen Activator
MCA: Middle Cerebral Artery
pMCAO: permanent Middle Cerebral Artery Occlusion
PBS: Phosphate Buffered Saline
ISOI: Intrinsic Signal Optical Imaging
LSI: Laser Speckle Imaging
